# Regulation of Pancreatic TXNIP-Insulin Expression Levels after Bariatric Surgery Using Diabetic Rodent Model

**DOI:** 10.1155/2023/9563359

**Published:** 2023-01-24

**Authors:** Jason Widjaja, Yuxiao Chu, Jian Wang, Jian Hong, Xiaocheng Zhu, Libin Yao

**Affiliations:** ^1^Department of Gastrointestinal Surgery, The Affiliated Hospital of Xuzhou Medical University, Xuzhou Jiangsu 221002, China; ^2^Department of General Surgery, Fudan University Affiliated Huadong Hospital, Shanghai 200040, China

## Abstract

**Purpose:**

The purpose of this study was to investigate the effect of bariatric surgery on pancreatic thioredoxin-interacting protein (TXNIP) and insulin expression levels. The research question is does bariatric surgery induce changes in the pancreatic TXNIP level, given that TXNIP has been proposed as a key glucose control factor?

**Methods:**

Using nondiabetic and diabetic rats, we investigated whether our streptozotocin-induced diabetic rat models exhibited changes in pancreatic TXNIP regulation. Following this confirmation, we randomly divided the diabetic rats into the following three groups: the gastric bypass group (*n* = 16), pair-fed group (*n* = 10), and sham group (*n* = 10). Preoperatively and 3 weeks postoperatively, all the rats underwent an oral glucose tolerance test, insulin tolerance test, and blood sampling procedures for hormonal analysis.

**Results:**

The TXNIP messenger ribonucleic acid (mRNA) and protein expression levels were significantly lower in the gastric bypass group than in the other groups. Regarding the gastric bypass group, the pancreatic mRNA expression levels of microRNA-204 (miR-204) and MafA were significantly lower and higher, respectively, than in the other groups. Furthermore, the levels of pancreatic insulin expression at the mRNA and protein levels were also significantly higher in the gastric bypass group than in the other groups.

**Conclusion:**

Bariatric surgery significantly improved glucose control and regulated the pancreatic insulin production pathways of TXNIP, miR-204, and MafA. The regulation of TXNIP, miR-204, and MafA might play an important role in the mechanism of diabetes remission following bariatric surgery.

## 1. Introduction

Bariatric surgery is known to have excellent outcomes for diabetes remission [1, 2]. However, the mechanism by which bariatric surgery induces diabetes remission remains unclear [2]. Although the concept of increased glucagon-like peptide-1 (GLP-1) levels post bariatric surgery is commonly used at present to explain diabetes remission, recent evidence has been controversial [3, 4]. However, few studies have shown that bariatric surgery induces improvements in glucose tolerance, even in GLP-1 receptor-deficient mouse models [5, 6].

Various other theories have also been proposed to explain the efficacy of bariatric surgery to induce diabetes remission. The changes in the bile acids, gut microbiota, and peptide tyrosine tyrosine (PYY) level have been proposed as possible postsurgery diabetes remission factors [7]. This notion led us to suspect that bariatric surgery-induced diabetes remission could be multifactorial and that there could be another possible factor yet to be investigated.

Thioredoxin-interacting protein (TXNIP) has been identified as a key factor in beta-cell biology and glucose homeostasis that is correlated with obesity [8–11]. It has been previously reported that TXNIP was upregulated in the diabetic pancreas and that low level of TXNIP was protective against type 1 and type 2 diabetes [12]. A previous study has also shown that pancreatic TXNIP regulates insulin secretion through microribonucleic acid- (RNA-) 204 (miR-204) and MafA regulation [13].

In a previous study of ours, we found that the plasma level of TXNIP was substantially increased in patients with obesity and diabetes. Furthermore, we found that bariatric surgery induced a significant reduction in postsurgery plasma TXNIP levels [14]. Currently, to the best of our knowledge, no other studies have investigated the relationship between bariatric surgery and pancreatic TXNIP levels. Therefore, this study is aimed at further investigating the early effects of bariatric surgery on pancreatic TXNIP regulation using diabetic rodent models.

## 2. Materials and Methods

### 2.1. Animals

This study was approved by the Ethics Committee for the Research in Animals at our institution. All applicable institutional and national guidelines for the care and use of animals in the People's Republic of China were followed.

Adult (8-10 weeks old, approximately 350 g in weight) male Sprague Dawley rats were obtained from our institutional Animal Research Centre. The temperature and humidity, with a 12 h day and night cycle, were maintained throughout the experiment. Diabetes was induced by an intraperitoneal injection of low-dose streptozotocin (STZ; 35 mg/kg dosage). The blood glucose level was first measured 72 hours after the STZ injection. The rats that had a random blood glucose level (measured through the tail vein) > 16.0 mmol/L for 3 consecutive days were considered to be diabetic.

### 2.2. Study Design

This experiment was divided into two steps. In the first step, we used normal (nondiabetic; *n* = 8) and diabetic rats (*n* = 8) to investigate whether changes in pancreatic TXNIP regulation in our diabetic rat models were exhibited. In the second step, after confirmation in our diabetic rat models, we induced diabetes in another 36 rats and randomly divided them into the following three groups: gastric bypass group (GB; *n* = 16), pair-fed group (PF; *n* = 10), and sham group (*n* = 10). The higher number of rats allocated to the GB group was to cater for the expected higher mortality rate following the surgical procedure. At 3 weeks postoperatively, 8 surviving rats from each group were randomly chosen and included in further experiments. Three-week postoperative period was chosen as significant blood glucose control improvements were observed 3 weeks following bariatric surgery.

### 2.3. Surgical Procedure and Food Intake

Concerning the second step of the experiment, all rats were fasted overnight before the surgery, and a sterile surgery was performed under anesthesia (1% pentobarbital sodium at a dosage of 4 ml/kg via intraperitoneal injection). A prophylactic antibiotic (ceftriaxone) was administered intramuscularly.

In the GB group, a small gastric pouch (approximately 20%) was created. The intestine used for the gastrointestinal anastomosis was measured approximately 30 cm away from the ligament of Treitz (resulting in one anastomosis GB model). The anastomosis was approximately 0.5 cm in width and sutured using 5–0 silk sutures (Ningbo Medical Needle Co., Ltd., Ningbo, China).

The PF and sham groups underwent similar control surgeries. A small opening (approximately 0.5 cm) was created on the stomach and intestine (30 cm away from the ligament of Treitz), which was then closed directly using 5–0 silk sutures (Ningbo Medical Needle Co., Ltd., Ningbo, China).

Their body weight and food intake were recorded preoperatively and up to 3 weeks postoperatively. The rats in the GB group received 1 g of normal dry chow on postoperative day 3, which was gradually increased each day until postoperative day 7. From postoperative day 8, the rats in the GB group received free access to normal dry chow; their daily intake was measured. The PF rats received the daily average quantity of food that was consumed by the GB rats on the same postoperative day throughout the experimental period. The rats in the sham group received 1 g and 3 g of normal dry chow on postoperative days 3 and 4, respectively; they then received free access starting from postoperative day 5.

### 2.4. Oral Glucose Tolerance Test (OGTT) and Insulin Tolerance Test (ITT)

OGTT and ITT were performed to investigate glucose utilization and insulin sensitivity/resistance. A 2 h OGTT and ITT were performed at 9 a.m. after overnight fasting before the operation and at 3 weeks postoperatively. The OGTT and ITT procedures were performed as described by Liu et al. [15]. The OGTT was performed with the gavage of a 50% glucose solution (3 mg/kg dosage), and the ITT was performed by intraperitoneal injection of human insulin (0.5 IU/kg dosage).

### 2.5. Blood Sample Collection and Hormonal Analysis

The blood samples for hormonal analysis (TXNIP and insulin levels) were obtained preoperatively and at 3 weeks postoperatively. The blood sampling was performed as described by Widjaja et al. [16]. To summarize, retroorbital blood samples were obtained once before and twice after (at 30 min and 60 min) oral gavage of 50% glucose solution (6 mg/kg dosage). The hormonal analysis was performed using an enzyme-linked immunosorbent assay kit (Bio-Swamp, Beiyinlai Biotechnology Co., Ltd., Wuhan, Hubei, China).

### 2.6. Quantitative Real-Time Polymerase Chain Reaction (RT-PCR) Analysis

At 3 weeks postoperatively, the rats from each group were ethically euthanized, and the pancreas specimens were collected and stored at −80°C until further use. For the RT-PCR tests, the total RNA from the pancreases of different rodent groups (GB, PF, and sham groups) was isolated using TRIzol (Thermo Fisher Scientific, 15596018, MA, USA), and cDNA was synthesized using HiScript® Q RT SuperMix (Vazyme Biotech Co., Ltd., R223-01, Nanjing, China) for RT-PCR analysis. The primer sequences used are shown in Table 1. The RT-PCR analysis was performed using iTaq® Universal SYBR Green SuperMix (Bio-Rad Laboratories, 1725121, Hercules, CA, USA) with the CFX Real-Time PCR System (Bio-Rad Laboratories, Hercules, CA, USA). The step cycling program used the following steps for a total of 40 cycles: denaturation at 94°C for 30 s, annealing at 94°C for 15 s, and extension at 60°C for 30 s. The expression levels of the pancreatic TXNIP, miR-204, insulin, and MafA were identified for the different rat groups.

### 2.7. Western Blot Analysis

For western blotting, the harvested pancreatic tissues were homogenized in a lysis buffer. The proteins were subjected to 10% sodium dodecyl sulphate-polyacrylamide gel electrophoresis (SDS-PAGE) and transferred to a polyvinylidene fluoride membrane. These membranes were incubated for 1 h at room temperature with 5% fat-free milk in a Tris-buffered saline that contained Tween® 20 (Sigma-Aldrich, St. Louis, MO, USA), followed by an overnight incubation at 4°C with the primary antibodies. The specific reactions were detected using rabbit horseradish peroxidase (HRP) conjugate secondary antibodies and were visualized using a Tanon chemiluminescence imaging analysis system (Tanon, Shanghai, China). The following antibodies were used: TXNIP (1 : 2000, Abcam, ab18865, Cambridge, UK) and INS (1 : 2000, Abcam, ab181547, Cambridge, UK).

### 2.8. Statistical Analysis

All data are presented as the mean values ± standard deviation (SD). The area under the curve (AUC) was calculated using GraphPad Prism 8 software for Windows (GraphPad Software, San Diego, CA, USA), using the trapezoidal method. A parametric one-way analysis of variance test with Bonferroni's multiple comparison test was used. A *p* value < 0.05 was considered statistically significant.

## 3. Results

### 3.1. Glucose Control and Pancreatic TXNIP-Insulin Expression Levels in the STZ-Induced Diabetic Models

**Figure 1:**
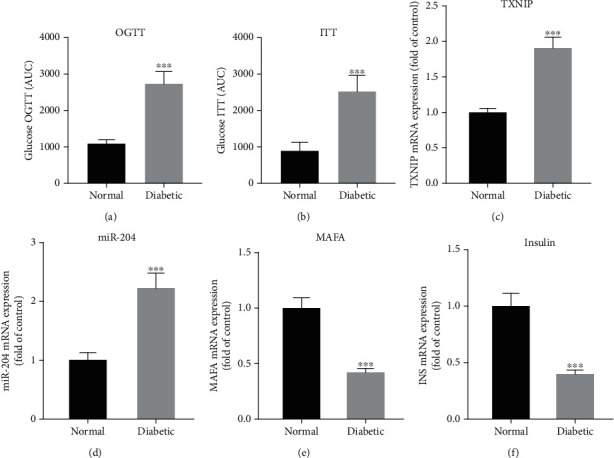
The difference in (a) oral glucose tolerance test (OGTT) and (b) insulin tolerance test (ITT) between normal and diabetic rats. The mRNA expression level difference in (c) TXNIP, (d) miR-204, (e) MAFA, and (f) insulin between normal and diabetic rats. ^∗∗∗^Significant between diabetic and normal (*p* value < 0.001). TXNIP: thioredoxin-interacting protein; miR-204: microRNA-204.

In the first step of the experiment, OGTT and ITT were performed for the normal (nondiabetic) and diabetic groups one week after STZ administration. The glucose AUC for the OGTT and ITT results showed significantly higher glucose levels in the diabetic group than in the normal group (*p* < 0.001; Figures 1(a) and 1(b)). The rats in both groups were euthanized one day after the OGTT and ITT were performed, and the pancreas was harvested for the RT-PCR analysis. The pancreatic mRNA expression levels of TXNIP, miR-204, MafA, and insulin were analyzed and compared. The mRNA expression levels of TXNIP and miR-204 were significantly higher in the diabetic group than those of the nondiabetic group (Figures 1(c) and 1(d)). On the contrary, the mRNA expression levels of MafA and insulin were significantly lower (Figures 1(e) and 1(f)) in the diabetic group than in the normal group (*p* < 0.001).

### 3.2. Body Weight and Food Intake in the Surgical Diabetic Models

**Figure 2:**
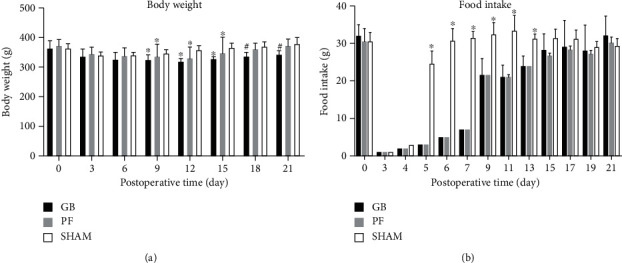
Change in (a) body weight and (b) food intake level among all the groups. All data are presented as mean ± standard deviation. ^∗^Significant GB and PF compared with sham (*p* value < 0.05). ^**#**^Significant GB compared with PF and sham (*p* value < 0.05). GB: gastric bypass; PF: pair-fed.

Concerning the second step of the experiment, no significant difference in the body weight and food intake of the GB, PF, and sham groups was observed preoperatively (Figures 2(a) and 2(b)). From postoperative days 9 to 15, the GB and PF groups showed significantly lower body weights than the sham group (*p* < 0.05). The body weight of the GB group was significantly lower than that of the PF and sham groups until postoperative day 21 (*p* < 0.05).

Regarding the food intake assessment, the sham group showed a significantly higher food intake from postoperative days 5 to 13 when compared to that of the other groups (*p* < 0.05). There was no significant difference observed in food intake among all groups from postoperative days 13 to 21.

### 3.3. The Glucose Control and Serum Levels of TXNIP and Insulin in the Surgical Diabetic Models

**Figure 3:**
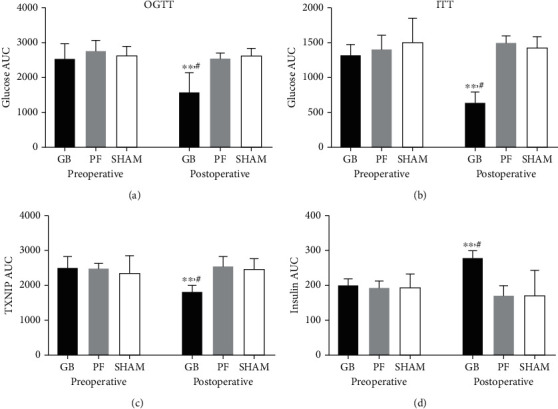
Preoperative and postoperative (a) oral glucose tolerance test (OGTT), (b) insulin tolerance test (ITT), (c) plasma TXNIP area under the curve (AUC), and (d) plasma insulin AUC between GB, PF, and sham rats. ^∗∗^Significant between preoperative GB and postoperative GB (*p* value < 0.01). ^**#**^Significant between postoperative GB compared with postoperative PF and sham (*p* value < 0.01). GB: gastric bypass; PF: pair-fed; TXNIP: thioredoxin-interacting protein.

Preoperatively, no statistical difference was observed in the OGTT and ITT results between the GB, PF, and sham groups (Figures 3(a) and 3(b)). However, 3 weeks postoperatively, significantly lower glucose AUC levels were observed in the GB group than those of the PF and sham groups (*p* < 0.01). For the TXNIP and insulin serum levels, no statistical difference was observed between the three groups preoperatively (Figures 3(c) and 3(d)). At 3 weeks postoperatively, the TXNIP and insulin levels were significantly lower and higher, respectively, in the GB group than those in the PF and sham groups (*p* < 0.01).

### 3.4. Pancreatic TXNIP-Insulin Expression Levels in the Surgical Diabetic Models

**Figure 4:**
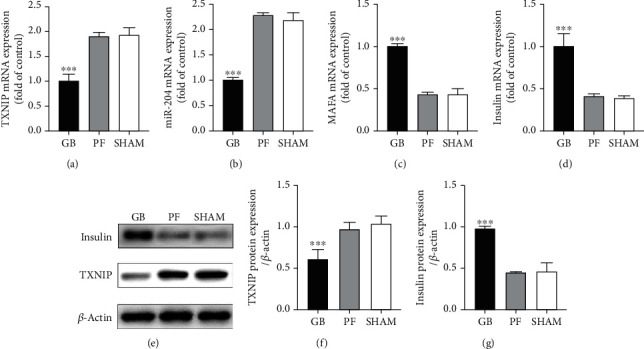
mRNA expression level in (a) TXNIP, (b) miR-204, (c) MAFA, and (d) insulin between GB, PF, and sham rats. (e) Representative western blots, (f) TXNIP protein expression level, and (g) insulin protein expression level between GB, PF, and sham rats. ^∗∗∗^Significant between GB compared with PF and sham (*p* value < 0.001). GB: gastric bypass; PF: pair-fed; TXNIP: thioredoxin-interacting protein; miR-204: microRNA-204.

Posteuthanasia and pancreas harvesting, the pancreatic mRNA expression levels of TXNIP, miR-204, MafA, and insulin were analyzed using the RT-PCR and compared. The postoperative mRNA expression levels of TXNIP and miR-204 were significantly lower (Figures 4(a) and 4(b)), and the expression levels of MafA and insulin were significantly higher (Figures 4(c) and 4(d)) in the GB group, when compared to those of the PF and sham groups (*p* < 0.001).

Western blotting was then used to analyze and compare the TXNIP and pancreatic insulin protein expression levels. The TXNIP protein expression level was significantly lower in the GB group, while the insulin protein expression level was significantly higher in the GB group than those of the PF and sham groups (*p* < 0.001; Figures 4(e)–4(g)).

## 4. Discussion

In this study, we showed that bariatric surgery has the potential to regulate the pancreatic TXNIP-insulin expression levels, which resulted in a significantly increased pancreatic insulin production. Furthermore, the expression levels of miR-204 and MafA, which are the key target factors in the regulation of the pancreatic TXNIP-insulin pathway [13], were regulated at three weeks following bariatric surgery. Therefore, we showed that bariatric surgery improved insulin secretion and glucose homeostasis, which is consistent with that reported in previous studies [17, 18]. Moreover, we showed the possibility that this is a result of the regulation of the pancreatic TXNIP, miR-204, and MafA levels.

The antidiabetic effect of bariatric surgery has become common knowledge; however, the underlying mechanistic reasons are still difficult to explain [1, 2, 19]. Conversely, TXNIP has been described as a potential master regulator of glucose homeostasis [20]. Studies in humans have shown that TXNIP is associated with diabetes and is able to regulate the peripheral glucose metabolism [21–23]. In vitro and in vivo studies showed that an overexpression of TXNIP resulted in beta-cell apoptosis and poor glucose control. Alternatively, a deficiency of TXNIP resulted in severe hypoglycemia and hyperinsulinemia and, as a result, is therefore considered to be protective against type 1 and type 2 diabetes [8, 12, 13]. Furthermore, an orally available chemical compound that can provide a distinct approach to treat diabetes with TXNIP regulation has been reported very recently [24].

TXNIP has been shown to control pancreatic insulin production through the TXNIP, miR-204, and MafA pathways [13]. The overexpression of miR-204 (which is expressed in both human and rodent pancreases) was found in TXNIP-overexpressing INS-1 cells, whereby the overexpression of miR-204 indirectly resulted in a >200% reduction in insulin mRNA expression levels [13]. Further analysis showed that only MafA, which is one of the key insulin transcription factors, was markedly reduced in response to the overexpression of miR-204. Therefore, this led to the increased production of pancreatic insulin through the TXNIP, miR-204, and MafA pathways [13].

Although bariatric surgery has been shown to improve glucose control in an STZ-induced diabetic rodent model [25–27], its effect on pancreatic TXNIP regulation has not yet been investigated. First, we confirmed that our STZ-induced diabetic rat model showed a significant elevation in pancreatic TXNIP expression levels. In addition to superior glucose control, bariatric surgery also resulted in significantly lower serum TXNIP and pancreatic TXNIP expression at the mRNA and protein levels. Regarding the bariatric surgery group, the miR-204 expression level was significantly lower, and the insulin transcription factor, MafA, was significantly higher than in the pair-fed and sham groups. A previous clinical study reported that the serum TXNIP levels were elevated in patients with obesity and diabetes and that bariatric surgery resulted in a significant reduction in serum TXNIP levels as early as 1 month postoperatively [14]. Therefore, our current animal study strengthens the notion that bariatric surgery induces rapid improvement in glucose control, possibly through the TXNIP regulation mechanism (the TXNIP, miR-204, and MafA pathways) [13]. However, further studies are needed to prove this hypothesis.

The current study showed interesting results; however, it also raised many issues that need to be solved. For example, there is the question of how bariatric surgery rapidly reduces TXNIP levels. GLP-1, an incretin that is most frequently discussed in relation to bariatric surgery, was reported to reduce TXNIP levels [28, 29]. However, the importance of GLP-1 in bariatric surgery remains controversial [5, 6]. There are limited studies on TXNIP and other possible gastrointestinal hormones such as glucose-stimulated insulinotropic polypeptide (GIP), oxyntomodulin, and PYY [19]. Therefore, future research is required to investigate the link between bariatric surgery and TXNIP levels.

If TXNIP is in fact the master regulator of glucose homeostasis, then a question that requires investigation is whether those postbariatric surgery patients with diabetes relapse showed a reelevated TXNIP level. Therefore, it is possible to utilize TXNIP as a biomarker for diabetes development.

The results of bariatric surgery are undisputed; however, there is still a lack of knowledge regarding the science of bariatric surgery. Although improving the technique of bariatric procedures is important for better and safer clinical results, it would be equally important to develop a better understanding of how and why bariatric surgery achieves these improved outcomes. We believe that bariatric surgery causes multifactorial effects, and it is possible that many other mechanisms are yet to be identified.

One of the limitations of this study is its small sample size. Furthermore, our current study focused only on the rapid improvements in glucose control following bariatric surgery. Long-term experiments to observe TXNIP levels and diabetes relapse after bariatric surgery are needed in the future. We acknowledged the need for validation study regarding the relationship between TXNIP and bariatric surgery.

## 5. Conclusion

Bariatric surgery significantly induced improved glucose control, lowered serum TXNIP levels, lowered pancreatic TXNIP and miR-204 expression levels, and elevated pancreatic MafA and insulin expression levels. The regulation of TXNIP, miR-204, and MafA might play an important role in the mechanism of diabetes remission following bariatric surgery.

## Figures and Tables

**Table 1:** The primer sequences used for the RT-PCR analysis.

TXNIP	Forward 5′-CAACTCAAGAGGCAAAGAA-3′
	Reverse 5′-AGTCAGCGTGGATGGAAA-3′
MafA	Forward 5′-CTGCTCCTCGGTGCCCTCTT-3′
	Reverse 5′-GGTGGTGCTGATACCCGCTCA-3′
INS	Forward 5′-TTTGTCAATCGGCATCTGTG-3′
	Reverse 5′-GGCGCTCCTTGGGTGTAT-3′
miR-204	Sequence 5′-TTCCCTTTGTCATCCTATGCCT-3′
	Forward 5′-CGGGCTTCCCTTTGTCATCC-3′
	Reverse 5′-CAGCCACAAAAGAGCACAAT-3′

TXNIP: thioredoxin-interacting protein; INS: insulin; miR-204: microRNA-204.

## Data Availability

The datasets generated during and/or analysed during the current study are available from the corresponding author on request.
